# Eruptive movements of ectopic permanent mandibular canines: a case series study based on serial orthopantomograms from ten children with unilateral ectopia and six children with bilateral ectopia

**DOI:** 10.1007/s40368-025-01049-y

**Published:** 2025-05-21

**Authors:** I. Kjær, M. Svanholt, P. Svanholt

**Affiliations:** 1 Department of Odontology, Faculty of Health and Medical Sciences, University of Copenhagen, Copenhagen, Denmark; 2 Section of Orthodontics, Department of Odontology, Faculty of Health and Medical Sciences, University of Copenhagen, Copenhagen, Denmark; 3 Copenhagen Municipal Clinic of Orthodontics, Copenhagen, Denmark; 4 Guldborgsund Municipal Clinic of Orthodontics, Nykøbing Falster, Denmark

**Keywords:** Eruption, Permanent canine, Primary canine, Mandible, Orthopantomogram

## Abstract

**Aim:**

The purpose of this study is to evaluate the patterns of eruptive movements of ectopic mandibular canines observed on series of orthopantomograms from each individual. The hypothesis is that the eruption direction might be predicted from the location and morphology of the permanent canine.

**Material and methods:**

Radiographic material was forwarded from orthodontic colleagues in Denmark and the Nordic countries. In 16 cases/individuals with unilateral or bilateral mandibular canine ectopia, more than one orthopantomogram from each individual was forwarded. The material consists of 41 radiographs from these 16 individuals. In each, the primary mandibular canine existed in the first taken orthopantomogram. The radiographs were taken with the same radiographic equipment, but not necessarily with the same settings and patient orientation. The material was divided according to unilateral ectopia (10 individuals) and bilateral ectopia (6 individuals), and according to the initial location of the permanent canine, compared to the axis of the primary canine (Ax) in the posterior location and anterior location. The following were registered: ages, lengths of age interval between orthopantomograms, canine maturity, root morphology, crown morphology, and location of permanent canines expressed in distances and angles.

**Results:**

The following were concluded. Ectopia was diagnosed earlier in bilateral cases (about 9 years of age) than in unilateral cases (about 12 years of age). More radiographs were taken for each child in the bilateral group, compared to the unilateral group. Extraction of the primary canines occurred often in the bilateral group, but seldom in the unilateral group. The posteriorly located unilateral ectopic canine was located further posterior, compared to the Ax, than bilateral cases. The crown morphology changed during the eruption movements. Curved root morphology appeared in the bilateral cases. Three different patterns were registered in canine eruption (upward, downward, and anterior movements), seemingly dependent on the location and crown morphology of the permanent canine. In bilateral cases, the eruption deviation was most severe on the left side.

**Conclusion:**

There are considerable differences between unilateral and bilateral ectopic permanent mandibular canines. The differences are predominantly in the age of the children when ectopia is diagnosed, the number of orthopantomograms taken, and furthermore the location, crown morphology and eruptive movements of the ectopic permanent mandibular canine.

## Introduction

Ectopic mandibular permanent canines are rare dental phenomena, discussed from different viewpoints.

The main purposes of these studies have been to elucidate the location, the occurrence, difference in gender, and possible treatments (Wertz 1994; Joshi 2001; Mupparapu 2002; Shapira and Kuftinec 2003; Aktan et al. 2010; Mazinis et al. 2012; Dalessandri et al. 2017; Plakwicz et al. 2019).

A problem which has received less attention is the question of etiology. Why and where have these canines started tooth formation? And why have they developed ectopically in different positions?

A stable structure for description and definition of canine ectopia has recently been defined (Kjær et al. 2023). This stable structure is the canine axis through the primary canine. The stability of the primary canine was based on histological studies of the primary canine and the surrounding alveolar process (Kjær and Bagheri 1999), which was different from the alveolar processes in the adjacent primary incisors.

Due to difficulties in early childhood for obtaining information on early sites for initial permanent canine analyses, early radiographs from children with arrested or absence of primary molars were studied (Kjær et al., 2023). This study revealed that the permanent bud of the mandibular canine has a normal position in the primary canine axis. In few cases, the permanent canines were located posterior to the primary canine axis, and in a single case, the permanent canine was located anterior to the canine axis (Kjær et al. 2023).

The study on mandibular canine positions (posterior or anterior to the primary canine axis) was followed up in a study based on single orthopantomograms from 47 individuals with ectopic mandibular canines (Svanholt et al. 2024). This recent study resulted in a subdivision of the orthopantomograms in three groups, demonstrating that the initial site of the permanent ectopic canine was in 6 cases located within the canine axis, in 36 cases posterior to the canine axis, and in 5 cases anterior to the canine axis. Accordingly, the primary canine axis seems useful for defining the location for early permanent canine formation. From this early stage in canine development, eruptive movements occur, but the direction of this eruption path needs to be studied in the analyses of series of orthopantomograms taken from the same individual over several years. Material for a study like that is difficult to obtain, because the ectopic canine in most cases is surgically removed, when first diagnosed. Also, the primary canine has often been extracted when the ectopic permanent canine was diagnosed.

The purpose of the present study is to evaluate patterns of eruptive movements of permanent ectopic mandibular canines observed on series of OPs from each individual.

The hypothesis is that the eruption direction (upward, downward, or anterior) might be predicted from the initial location and morphology of the permanent canine.

## Material and methods

### Material

The material consists of a series of orthopantomograms from each child or young adult, diagnosed with ectopic mandibular canines. This material was forwarded from orthodontic colleagues in Denmark and the Nordic countries over a period of 24 years (1998–2022). In 16 patient cases, diagnosed with mandibular canine ectopia, more than one orthopantomogram from each individual was forwarded. The material in the present study consists of 41 orthopantomograms taken at different ages from each of these 16 patient cases. The radiographs have been taken with the same radiographic equipment, but not necessarily with the same settings and patient orientation.

The material was divided into unilateral ectopic cases and bilateral ectopic cases. The material was also divided according to registration of initial sites for permanent canine development (Kjær et al 2023; Svanholt et al. 2024). This registration divided the material into: cases where the initial sites of the permanent canines were located posteriorly or anteriorly to the axis of the primary mandibular canine axis (Ax).

#### Posterior initial site

In the collection, where the initial site was a posterior location of the early permanent tooth bud, two different groups appeared.

*The unilateral group.* This is a group in which posterior canine ectopia was registered only on one side of the mandible, resulting in orthopantomograms from nine individuals for registration. In this group, five cases demonstrated canine ectopia in the patients’ left side and four cases in the patients’ right side. For easy comparison of the orthopantomograms, the radiographs were turned horizontally, so the ectopic mandibular canine was always presented on the same side in the figures. Two orthopantomograms were available from each individual.

*The bilateral group.* This is a group in which ectopic mandibular canines were registered on both sides of the mandible, resulting in six patient cases with 12 ectopic regions for registration of canine ectopia. Three to five orthopantomograms were available from each individual.

#### Anterior initial site

In the collection, ectopic permanent mandibular canine was registered unilaterally, anteriorly located, in one case.

To sum up, 22 mandibular regions with ectopic mandibular canines were described in this study. 12 regions were from bilateral cases, and 10 regions from unilateral cases. Of the 22 regions, 21 regions were from children with initial sites for canine positions located posteriorly compared to the primary canine axis Ax. In one child, the onset of canine formation started anteriorly to the primary canine axis, Ax.

### Methods

The following parameters were registered:

*Ages:* Age (if available) for the first orthopantomogram was listed in the three groups: The unilateral posterior group, the bilateral posterior group, and the anterior group.

*Lengths of age interval:* The time interval (if available) between the orthopantomograms of each child was listed.

*Canine maturity:* The maturity of the ectopic canine was registered in each orthopantomogram, OP: 0 = crown completed, 1 = 1/3 root completed, 2 = 1/2 root completed, 3 = 2/3 root completed, 4 = root almost completed with open apex, and 5 = root apex completed.

**Fig. 1:**
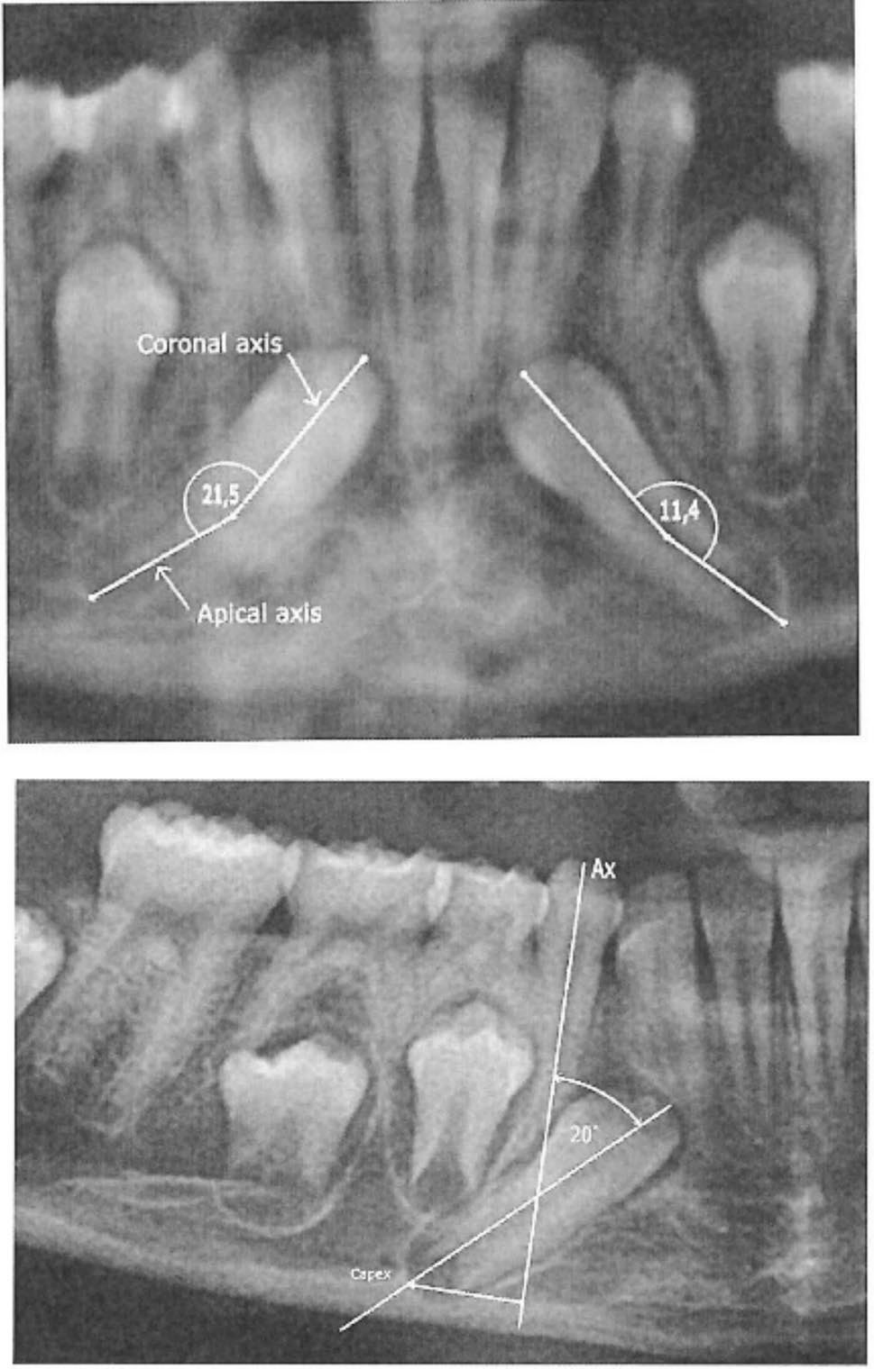
Upper: a section of an orthopantomogram illustrating bilateral ectopic mandibular canines. Marked by white lines are coronal axis drawn from the cusp to the estimated midpoint of the root and the apical axis between the midpoint of the root to the canine apex. The angle between these two lines expresses the root morphology described as straight (S) or curved (C). If this angle is larger than 10 degrees, the root morphology is expressed as curved. In this bilateral case, the angles are 21,5 degrees and 11,4 degrees, both indicating curved root morphology C. Lower: a section of an orthopantomogram illustrating the location of a left sided unilateral ectopic mandibular canine. The OPG has been flipped horizontally so that the ectopic canine appears at the right side. The Ax line marked illustrates the axis of the primary canine. The location of the permanent canine is expressed as the distance from the axis of the primary canine, Ax and as the angle between the permanent and primary mandibular canines. The distance from the permanent canine apex (C apex), estimated on a line perpendicular to Ax, expresses the posterior location of the permanent canine compared to Ax. Changes in this distance during development express movements of the permanent mandibular canines mainly in the horizontal plane. The angle between Ax and the axis marked in the ectopic permanent mandibular canine from the cusp to apex indicates the angulation of the permanent canine 20 degrees measured on the actual radiograph. Changes in angulation during development indicate downward (angle enlarged) and upward (angle diminished) movement of the permanent mandibular canine

*Root morphology:* Root morphology describes the curvature of the root in the permanent ectopic canine, as demonstrated in Fig. 1, upper part. The curvature is defined as the angulation between the coronal axis of the ectopic canine and the apical axis. The coronal axis is drawn between the cusp and the estimated midpoint of the root. The apical axis is the line between the midpoint of the root and the apex. The curvature is registered as C (curved), when the angle between the axes is larger than 10^◦^ and as S (straight) when the angle is less than 10^◦^.

*Location:* The location of the permanent ectopic canine is described according to the distance from the permanent canine apex, estimated on a line perpendicular to the primary canine axis Ax, and according to the angle between the axes of the permanent and primary canines (Fig. 1 lower). Changes in distances during development express movements of the permanent mandibular canines mainly in the horizontal plane. Changes in angulation indicate downward (angle enlarged) and upward (angle diminished) movement of the permanent mandibular canine.

**Fig. 2:**
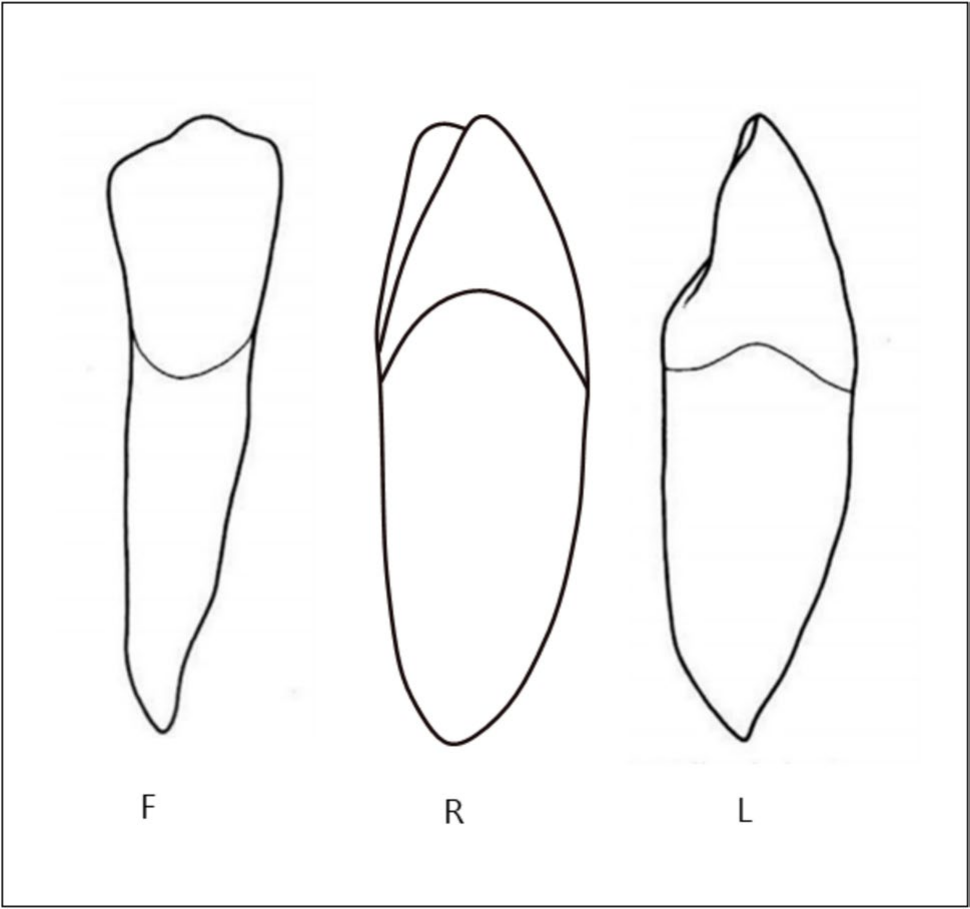
Schematic drawings illustrating the morphology of the permanent mandibular canines in the facial view (F), lateral view (L) and rotated view (R). The drawing of the rotated permanent canine is shown in a developmental stage between F and L

*Crown morphology:* The crown morphology was described according to a modification from Svanholt et al. (2020) in F, facial appearance on orthopantomogram, R, rotated appearance on orthopantomogram, and L, lateral appearance on orthopantomogram (Fig. 2).

The root and crown morphology and the location are estimated using View Box 3® (Demetrios Halaznetis, Athens, Greece).

## Results

### Posterior initial site

#### Unilateral group

**Fig. 3:**
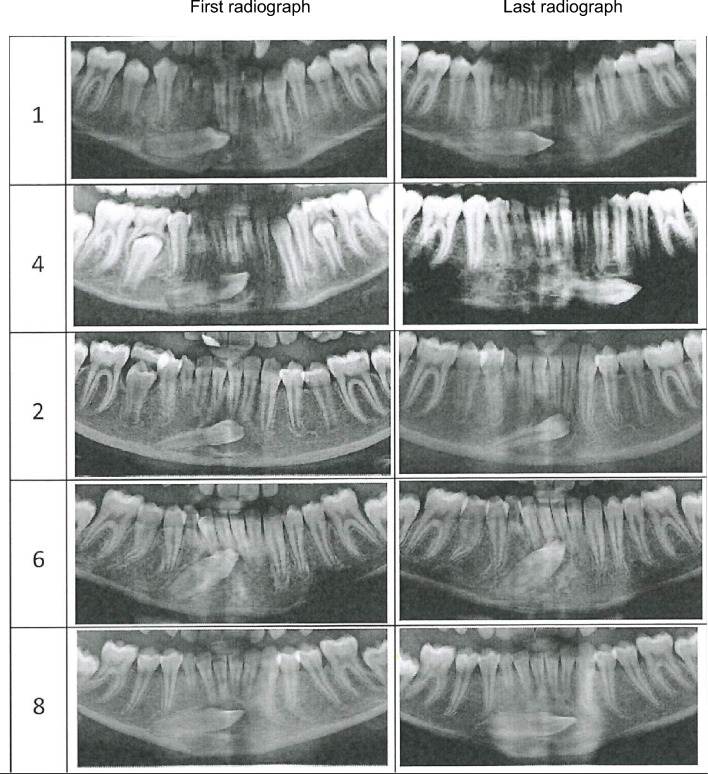
Overview of five unilateral ectopic permanent mandibular canines initially posteriorly located compared to the axis of the primary canine. The cases in the figure are 1, 4, 2, 6, and 8 listed in Table 1. The five cases are arranged according to the initial maturity scores of the mandibular canines from score 3 (Case 1) to score 5 (Case 8). In all cases, two orthopantomograms were available, marked as first radiograph and last radiograph. The orthopantomograms were flipped horizontally to appear on the right side in the five cases because the unilateral ectopic canines occurred on the left side in these cases. The differences in crown morphologies are highlighted in the following: Case 1: Initial crown morphology: Rotated. The eruptive movement of the canine during 12 months was slightly in an anterior and downward direction. In the second radiograph, the crown had attained a lateral morphology. Case 4: The initial crown morphology: Lateral. The eruptive movement of the canine during 36 months seems to be in the anterior and downward direction. In the second radiograph, the crown still has a lateral morphology. Case 2: Initial crown morphology: Facial. The eruptive movement of the canine during 12 months is slightly anterior and in anupward direction. In the second radiograph, the crown on the canine still has a facial appearance. Case 6: Initial crown morphology: Rotated. The eruptive movements of the canine during 12 months are slightly anterior and slightly upward. In the second radiograph, the crown on the canine still has a rotated appearance. Case 8: Initial crown morphology: Lateral. The eruptive movements of the canine during 11 months are in a slightly anterior and downward direction

The results are illustrated in Table 1 and Fig. 3.

**Table 1:** Unilateral ectopia of mandibular permanent canines/initial site, posterior

Case	OP side	Age		Maturity	Morphology		Location		Dentition
		First OP	Interval		Root	Crown	Apex rel to Ax,mm	Angulation degrees	
1a	Left	11^2^		3	S	R	9.8	80.6	–
1b			12	3	S	L	8.5	93.3	
2a	Right	11^8^		4	S	F	8.5	76.8	–
2b			12	5	S	F	6.4	74.9	
3a	Left	11^10^		5	S	L	6.7	93.2	Taurodontia of the second maxillary molars, agenesis of maxillary third molars, ectopia of second maxillary molar
3b			18	5	S	L	6.0	85.2	
4a	Left	12^0^		3	S	L	11.0	80.2	Taurodontia of second maxillary molars
4b			36	5	S	L	– 6.5	99.8	
5a	Right	12^3^		4	S	R	11.9	75.3	Agenesis of third mandibular molar
5b			14	5	S	L	–	87.2	
6a	Left	12^11^		4	S	R	8.8	64.7	–
6b			12	5	S	R	7.1	63.0	
7a	Right	a		4	S	L	3.7	64.1	Agenesis of third maxillary molars, agenesis of third mandibular molars and second mandibular premolars, ectopia of second premolars and one canine in the maxilla
7b			8	5	S	L	2.7	64.6	
8a	Left	–		5	S	L	12.2	86.6	Taurodontia of second maxillary molars
8b			11	5	S	L	10.3	98.5	
9a	Right	–		1	–	F	10.7	50.8	–
9b			18	1	–	F	4.7	22.2	

Overview of nine unilateral ectopic mandibular canines initially posteriorly located, compared to the axes of the primary canine (Ax). Five of these cases are demonstrated in Fig. 3. This table demonstrates that two radiographs (a and b) are available in each patient case, marked with number. Ages between the radiographs are noted. Maturity of root development is observed from stage 1–5. S indicates straight roots, F indicates crown with facial morphology, R indicates rotated morphology, and L indicates lateral appearance. The location of the ectopic mandibular canine is noted as the distance from the permanent canine apex to the Ax, and angulation is measured as the angle between the ectopic permanent mandibular canine and the primary canine

*Ages:* In the nine patient cases registered, the ectopia was first diagnosed on orthopantomograms at the ages between 11 years, 2 months and 13 years, 7 months. In two cases, the ages were not available.

*Numbers of orthopantomograms:* In all cases, the numbers of existing orthopantomograms were two, and the time interval between the two orthopantomograms was from 8 to 36 months.

*Canine maturity:* The maturity of the canines, when first registered, was from the initial stage of tooth formation (1 case) until complete root formation (3 cases).

*Morphology:* The morphology of the roots was in all cases straight and the crown morphologies appeared initially either with facial, lateral, or rotated appearances.

*Location:* The locations and movements of the five cases are demonstrated in Fig. 3. These are cases no. 1, 4, 2, 6, and 8 (Table 1).

Concerning the locations of the permanent canine, the distance observed between the permanent apex and the Ax indicated that the canines initially were severely distally located in a close relation to the root of the first premolar. In all cases, this distance diminished from the first to the second radiographs. These changes in distances indicate anterior movement of the ectopic permanent canine. In one case where the crown had a lateral appearance (case 4 Table 1), the canine moved 17.5 mm anteriorly for 36 months.

The angulation between the primary canine and the permanent canine also changed during the observation period. In most cases, this angle enlarged, resulting in a direction of the permanent canine pointing more downward. This is what appeared in case 4 (Tabel 1, Fig. 3), where the angle between the axis of the permanent and primary canines increased from 80.2 degrees to 99.8 degrees. The initial crown morphology in case 4 was lateral L. In case 6 (Table 1, Fig. 3), the permanent canine moved less than 1 mm anteriorly for 12 months and the canine moved slightly upward. In this case, the canine had the initial maturity score 4, and the crown morphology was rotated, R. In case 2 (Table 1, Fig. 3), the crown had facial appearance F, and the movement of the canine for 12 months was slightly upward.

In the five cases demonstrated in Fig. 3, the initial crown morphologies were facial (1 case) rotated (2 cases), and lateral (2 cases). In the cases demonstrated, the initial maturity of the canines was three in two cases, four in two cases, and five in one case.

Figure 3 focuses on the importance of the morphology of the permanent canine crown for predicting the eruptive pattern. Canines with initial lateral appearances seem to move downward, but an extended study with more material is needed for confirmation. Table 1 indicates that upward movement of the canine toward the occlusal plane (lowering of the angle between the primary and permanent canine) does not occur when the canine on OPs appears in a rotated or lateral position. The importance of the canine maturity for prediction of the eruptive movements cannot be concluded.

*General dentition:* Dental deviations are observed in the dentition. These are taurodontia and agenesis in five of the nine cases (including one case with agenesis of the third molars).

#### Bilateral group

**Fig. 4:**
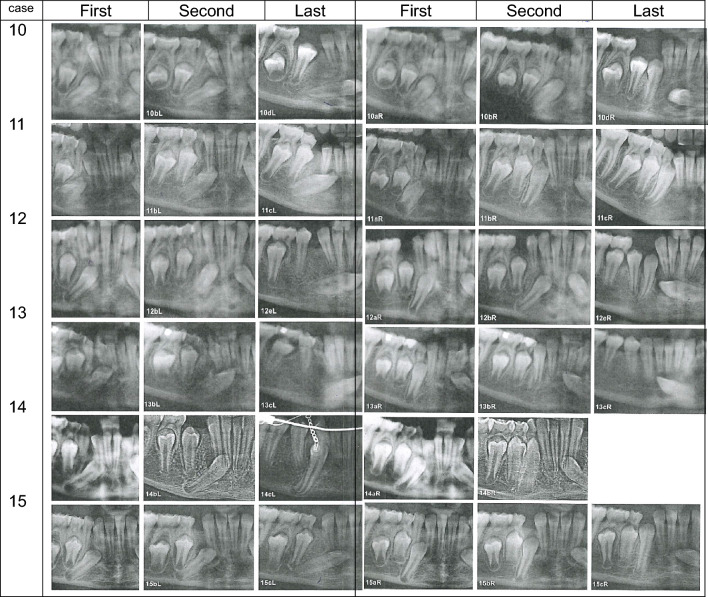
Overview of the bilateral ectopic mandibular canine regions from the six cases presented in Table 2 (Case 10–15). In cases where the ectopic canine occurred on the left side, the orthopantomogram was flipped horizontally so that all cases studied appeared on the right side. The left ectopic cases in the individual are presented to the left in the figure (first, second, last) and the right ectopic cases in the individual (first, second, last) are presented turned horizontally and appear to the right.Three radiographs appear in four cases, four radiographs in one case, and five radiographs in one case. **Comparison of the left and right side** *Crown morphology initial:* The initial crown morphology in the right side of the Figure has facial appearances in all six cases. In the left side of the Figure, facial and rotated initial morphology appears. *Canine maturity initial:* Canine maturity seems to be close to identical in the two sides. *Root morphology:* Curved morphology seems to be more frequent on the left side of the Figure compared to the right side, but with small deviations. Eruptive movements: In the left side, the canines are seemingly not moving upward, as the angulation degrees increase. In the right side, all canines seem to move upward. As a conclusion, there is a difference between the left and right regions of ectopic bilateral canines. Canines in the right region of the individuals seem to move occlusally compared to canines in the left regions. *General dentition:* Deviations in the dentition are observed in five of the six cases with bilateral canine ectopia, but no deviations are observed in case 15

The results are illustrated in Table 2 and Fig. 4.

**Table 2:** Bilateral ectopia of mandibular permanent canines/Initial site, posterior

Case	Age		Maturity		Morphology				Location				Dentition
					Root		Crown		Apex rel to Ax,mm		Angulation degrees		
	First OP	Interval	Left	Right	Left	Right	Left	Right	Left	Right	Left	Right	
10a	8^0^	–	1	1	S	S	F	F	3.1	4.1	40.6	32.9	Agenesis of all third molars and both maxillary second incisors
10b	–	–	2	2	S	S	F	F	2.1	2.6	48.0	37.1	
10c	–	–	3	2	S	S	F	F	–	–	–	–	
10d	–	48	4	4	S	S	R	R	–	–	–	–	
11a	8^4^		0	0	–	–	R	F	7.8	4.8	48.5	29.8	Agenesis of all third molars, taurodontia of maxillary second molars
11b	–	36	2	2	–	S	R	F	6.6	5.2	51.0	24.4	
11c	–	12	3	3	–	S	L	F	–	2.7	–	13.3	
12a	9^0^		2	2	S	S	F	F	5.4	8.7	35.3	49.4	Taurodontia of maxillary second molars
12b	–	11	2	2	S	C	F	F	8.2	8.3	53.4	53.3	
12c	–	10	3	2	C	S	F	R	–	–	–	–	
12d	–	5	4	3	C	S	F	R	–	–	–	–	
12e	–	12	5	4	C	S	F	L	–	–	–	–	
13a	9^0^		2	2	S	S	R	F	4.4	3.7	51.8	15.4	Taurodontia of maxillary second molars
13b	–	13	2	4	S	S	R	F	2.6	-	55.5	–	
13c	–	24	5	5	S	S	L	F	–	-	55.7	–	
14a	9^0^		2	2	C	C	F	F	5.6	6.8	32.2	47.8	Diminutive lateral maxillary incisor
14b	–	–	3	3	S	C	R	F	3.5	–	11.8	–	
14c	–	22	5	4	S	C	L	F	–	–	–	–	
15a	10^0^		2	2	C	S	F	F	6.1	3.1	44.1	19.8	
15b	–	12	3	4	C	S	F	F	–	–	–	–	
15c	–	12	4	4	S	S	R	F	–	–	–	–	

Overview of the bilateral mandibular canine regions from the six cases illustrated in Fig. 4. The cases are marked 10–15, as continuation numbers from Table 1. From each case, three to five radiographs were investigated. The individual radiographs are marked a, b, c, d, e. The interval indicates the time from the first to the last radiograph. Left and right indicate the left side (region) of the mandible and right side (region) of the mandible. S indicates straight morphology of the root, and C, curved morphology. The crown morphology is defined as F (facial appearance), R (rotated appearance), and L (lateral appearance). The location of the ectopic permanent mandibular canines is noted as distance from the permanent canine apex to the Ax, and angulation is measured as the angle between the permanent mandibular canine and the primary mandibular canine

*Ages:* In the six patient cases registered, the ectopia was first diagnosed on orthopantomograms at the ages between 8 years, 0 months and 10years, 0 month.

In all six cases, the ages were available.

*Number of orthopantomograms:* The numbers of orthopantomograms in each case were from three to five radiographs (Table 2), and the time intervals between the orthopantomograms taken were from 5 to 48 months.

*General dentition:* General dental deviations are observed in the dentition, such as taurodontia, agenesis in four of the six cases, and in one case diminutive lateral maxillary incisors appeared. In only one case other dental deviations (except canine ectopia) did not occur.

*Subdivision of the bilateral groups.* As all the six cases appeared with bilateral mandibular canine ectopia, 12 regions of ectopic canines were registered. Accordingly, distinctions between the left and right appearances in patients could be established. The findings are presented on the left side and right side are shown in Fig. 4.

##### Bilateral ectopia, individual’s left side

Maturity: The canines, when first registered, were immature, from the initial stages of root formation 0–2.

Morphology: The morphology of the roots was in most cases straight, but also curved morphologies occurred. In two cases, the initial morphology was curved. The initial crown morphologies had facial, lateral, or rotated appearances.

Locations: Concerning locations of the ectopic permanent mandibular canine, the distance between the permanent apex and the Ax axis indicated that the canine was distally located, but not in close relation to the root of the first premolar. In cases where information was available, this distance diminished from the first to the second radiograph. These changes in distances indicate anterior movement of the ectopic mandibular canine. In one case where the crown had a facial appearance (case 10, Table 2), the canine moved anteriorly and the angulation increased, indicating that the canine moved downward. The crown morphology changed during 48 months from F to R.

The angulation changed during the observation period in most cases to a direction pointing more downward. In case 14, the opposite was observed. For 22 months, the angle seemed to diminish. This improved the possibility of orthodontic treatment.

##### Bilateral ectopia, the individual’s right side

*Maturity:* The permanent canines, when first registered, were immature from the initial stages 0–2.

*Morphology:* The morphology of the roots was in three cases straight and in one case straight in the beginning and later curved. In one case, the initial morphology was curved. The permanent crown morphologies appeared with facial appearance in all five cases and only in two cases the morphology changed from facial to rotated and ended up with lateral or rotated appearances.

*Locations:* Concerning locations of the permanent canine the distance between the permanent apex and the axis of the primary canine, Ax axis indicated that the canine was distally located but not in close relation to the root of the first premolar. In all cases observable, this distance diminished from the first to the second radiographs. These changes in distances indicate anterior movement of the ectopic canine. In one case where the crown had a facial appearance (case 11, Table 2), the canine moved anteriorly during 36 months, and the angle diminished, resulting in an upward movement.

The angulation: It also changed during the observation period in most cases to a direction pointing more upward. This is what appeared in case 11 (Tabel 2), where the angle between the axis of the permanent and primary canines diminished from 29.8 degrees to 13.3 degrees, indicating an upward movement. In case 15, the same was observed (not measured) on orthopantomograms after 12 months.

*Specific comments:* One interesting finding is the initial maturity of the ectopic canines in the bilateral case 11. These canines initially had a bilateral maturity index or close to 0, meaning that only the crown had formed. The crowns was tilted mostly in the individual’s left side. From this initial tilted position, the canine erupted ectopically downwards. This was not what appeared int he right side of the individual. It is obvious that the ectopic tilted position of the crown can predict ectopic eruption.

### Anterior initial site

**Fig. 5:**
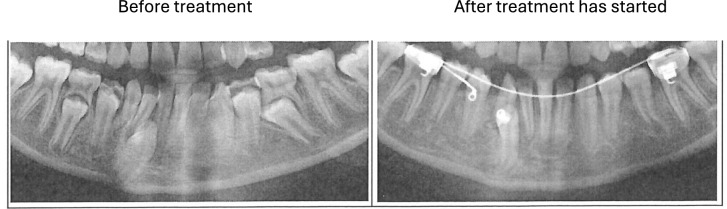
Overview of unilateral ectopic permanent mandibular canine (Case 16) with the orthopantomogram flipped horizontally to the left, so that the ectopic canine appears on the right side. The canine was initially anterior located compared to the axis of the primary canine (Ax), presented in Table 3. This case is documented before orthodontic treatment and after orthodontic treatment had started. The initial position of the ectopic canine is between the permanent lateral and permanent central mandibular incisors. The primary canine on the left side has been extracted and the orthodontic treatment had not been completed

The results are illustrated in Table 3 and Fig. 5.

**Table 3:** Unilateral ectopia of mandibular permanent canines/Initial site, anterior

Case	Age		Maturity	Morphology		Location		Dentition
	First OP	Interval		Root	Crown	Apex rel to Ax, mm	Angulation degrees	
16a	–	–	2	C	L	– 1	16.9	Taurodontia of the maxillary second molars, ortho treated primary canine extracted, ortho appliance inserted
16b	–	–	5	C	F	–	–	

Overview of the single case with unilateral left ectopic mandibular canine initially located anterior to the Ax. The orthopantomogram was flipped horizontally to the left so that the ectopic canine appeared to the right. Two radiographs are demonstrated, marked a and b. Maturity scores in the first radiograph is 2, and in the last radiograph 5. C indicates curved roots. L and F indicate lateral and facial crown morphology of the permanent mandibular canine

One case (case 16) belongs to this group, as demonstrated in Table 3. The first orthopantomogram confirmed the onset of formation of the permanent canine anterior to the primary canine axis Ax. The angle between the permanent and primary canines was 16.9, which indicated that the permanent canine had the ability to erupt toward the occlusal plane. The primary canine was extracted, and an orthodontic appliance inserted. Radiographs after orthodontic treatment did not exist.

### Similarities/differences between groups

When the unilateral and bilateral cases were compared, the following can be concluded:

1. Ectopia of the permanent mandibular canine is diagnosed earlier in bilateral cases than in unilateral cases.
2. The position of the apex from the posteriorly located unilateral ectopic canine is located further posterior, compared to the same distance in bilateral cases.
3. The crown morphology often changes during the eruption period in both groups.
4. Extraction of the primary canines occurs in the bilateral group, but seldom in the bilateral group.
5. Number of orthopantomograms: a greater number of orthopantomograms are taken for each child in the bilateral group, compared to the unilateral group.
6. Curved root morphology appears only in the bilateral group.
7. Canine eruption movements: There seems to be two different patterns in canine eruption. Canines moving upward have a more stable location compared to the first premolar, while canines moving downward move away from the first premolar. Furthermore, there seems to be an association between permanent crown appearance and eruption directions and a striking difference in the eruption pattern in the right and left sides in the bilateral cases. The left side has more eruption deviations compared to the right side.

## Discussion

Ectopic mandibular canine location is a rare condition. The present study reveals new information concerning differences in unilateral and bilateral ectopia of the permanent mandibular canine. The differences are that the bilateral cases are diagnosed several years before the unilateral cases.

Unilateral ectopia was diagnosed in children about 12 years of age, while bilateral ectopia was diagnosed in children about 9 years of age. During the observation periods, a greater number of orthopantomograms were taken in bilateral cases, compared to unilateral cases.

Movements of the ectopic permanent mandibular canines have seemingly not been analyzed systematically before. It is important information for this study that the preceding primary canine has a staple location from where the movements of the permanent canine can be expressed.

As movements on orthopantomograms are difficult to express, there are several limitations in a study like the present.

These limitations are:

1. Difficulties in obtaining useful materials for a study like the present are that the primary canines often are extracted already in the first orthopantomogram available. The indication for this extraction was to improve the eruption of the permanent canine. This extraction prohibits construction of the stable structure Ax, which is necessary for analyses.
2. Another limitation in obtaining material for a study like the present is that the ectopic permanent mandibular canine is often surgically removed when first diagnosed. This condition also prevents analyses as described in the present study.
3. Gathering radiographic materials from different clinics results in differences in radiographs, patient orientation, and exposure time when cases are compared.
4. The inequality in radiographic material results in difficulties in comparison of measurements such as distances and angles. These parameters depend on radiographic exposure and the position of the patient. Therefore, only severe changes in these measurements are mentioned in the text.
5. Canine maturity depends on the estimation of the midpoint of the root of the developing permanent canine. This is an uncertain estimation.
6. Also the orientation of the canine in the transverse plane makes comparison inaccurate. The transverse plane has not been studied in this present study.
7. Distortion of structures during radiographic exposure is also a parameter making comparative studies of orthopantomograms difficult. This concerns a precise evaluation of crown morphology, and also the curvature of the roots in the permanent canines raise questions: Are the canines curved during root formation because they are immature and have “tried” to change eruption direction toward the occlusal plane? This is a question for the future.

In the present study, several differences have been observed between unilateral and bilateral ectopic canine cases: worth mentioning are ages and canine maturity, when first diagnosed. The bilateral cases are diagnosed 2–3 years before the unilateral cases.

General dental deviation in the dentitions appears in unilateral as well as in bilateral cases. Association between dental deviations such as agenesis, malformations, and ectopia have formerly been described (Kjær 2014, 2017a, b). The etiology behind this might be the nature and composition of the dental lamina formed from the ectoderm. Dental deviations are often interrelated with epithelial defects in the tooth lamina, and this results in several different dental deviations.

The permanent tooth bud can be laid down in a rotated position at the initial site and cause the deviation in crown morphologies and eruption direction. In a recent publication (Kjær et al. 2023), mandibular canines were diagnosed early in an ectopic position before age 6. It is worth mentioning that case 11 in the present study, of a child, 8 years and 4 months old, with initial maturity stage 0 (only crown formed) during the follow-up radiographs, demonstrated severe ectopic eruption.

There seems to be an interrelationship between the initial location of the ectopic permanent mandibular canine and the eruption path. This interrelationship seems to be associated with morphological deviations in the morphology of the crown of the ectopic canine. A study based on more material is needed for proving these associations.

It is worth noting that transposition of canines and first premolars in the maxilla is combined with skeletal maxillary retrognathia in bilateral cases, but not in unilateral cases (Danielsen et al., 2015). Another study on 75 children and young adults with non-syndromic, palatal displaced maxillary canines, performed by Shirazi and Kjær (2018), revealed that the etiology behind palatal unilateral and palatal bilateral maxillary canines is different. The unilateral cases seem to have a dental etiology, while the bilateral cases seem to have a skeletal etiology. This indicates that the etiology behind bilateral ectopia of the mandibular canines has a skeletal etiology, while unilateral ectopia has a dental etiology. This should in the future be elucidated by cephalometric measurements on frontal radiographs and profile radiographs.

Prediction, prevention, and treatment of ectopic mandibular canines are still raising questions.

From the present study, it might be concluded that dentitions with several dental deviations might be exposed also for ectopic development of the permanent mandibular canines. The present study focuses on differences in unilateral and bilateral permanent mandibular ectopia. The location of the permanent mandibular canine, compared to the primary canine axis and different morphology of the crown of the permanent canine, might improve the understanding and prediction of the eruption path and treatment possibilities of ectopic mandibular canines. These aspects need to be further studied.

## Conclusion

From serial orthopantomograms, the patterns of eruptive movements of ectopic permanent mandibular canines can be evaluated from the initial location of the permanent canine in the mandible. The initial location is described in relation to the Ax of the preceding primary canine which is a stable structure.

The eruption pattern from the initial location can be expressed by measuring the distance from the apex of the permanent canine to Ax, and measuring the change in angulation between Ax and the axis of the permanent canine. Also, observation of change in crown morphology of the permanent canine can add important information to eruption patterns.

The present study has revealed significant differences in the eruption pattern observed in unilateral and bilateral cases with ectopic permanent mandibular canine.

The posterior initial sites for the unilateral ectopic canines were located more posterior than the initial sites for formation of the bilateral canines.

From the posterior initial sites of a permanent canine, the eruptive movements start. From here, several canines have a downward eruption pattern, but few move in an upward direction.

The eruption pattern seems to be associated with the morphological appearance on orthopantomogram of the permanent crown of the canines. The morphology of the crown, expressed as facial, rotated, or lateral appearance, often changes during eruption.

In the bilateral group, the permanent canines in the right side appeared initially with facial morphology of the crown. All cases in the right side had the ability to erupt into the dental arch. This was not observed on the left side.

## Data Availability

Due to the nature of the research, supporting data is not available.
